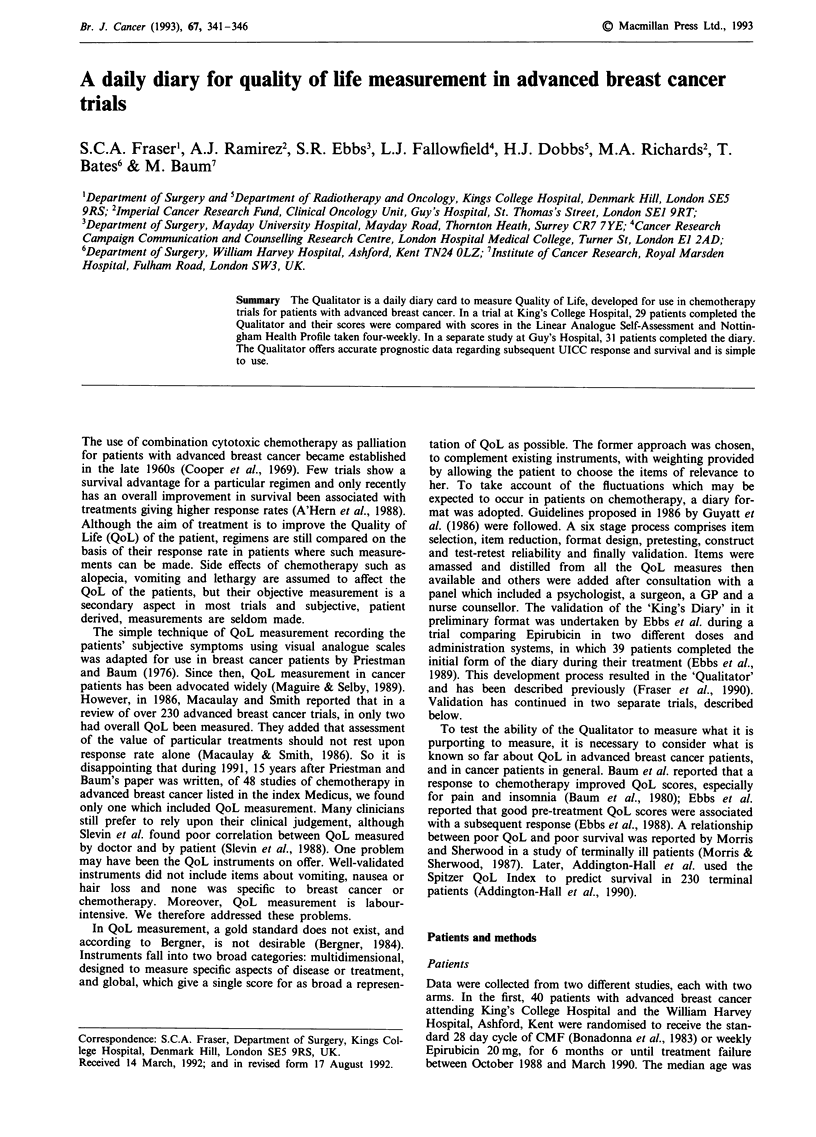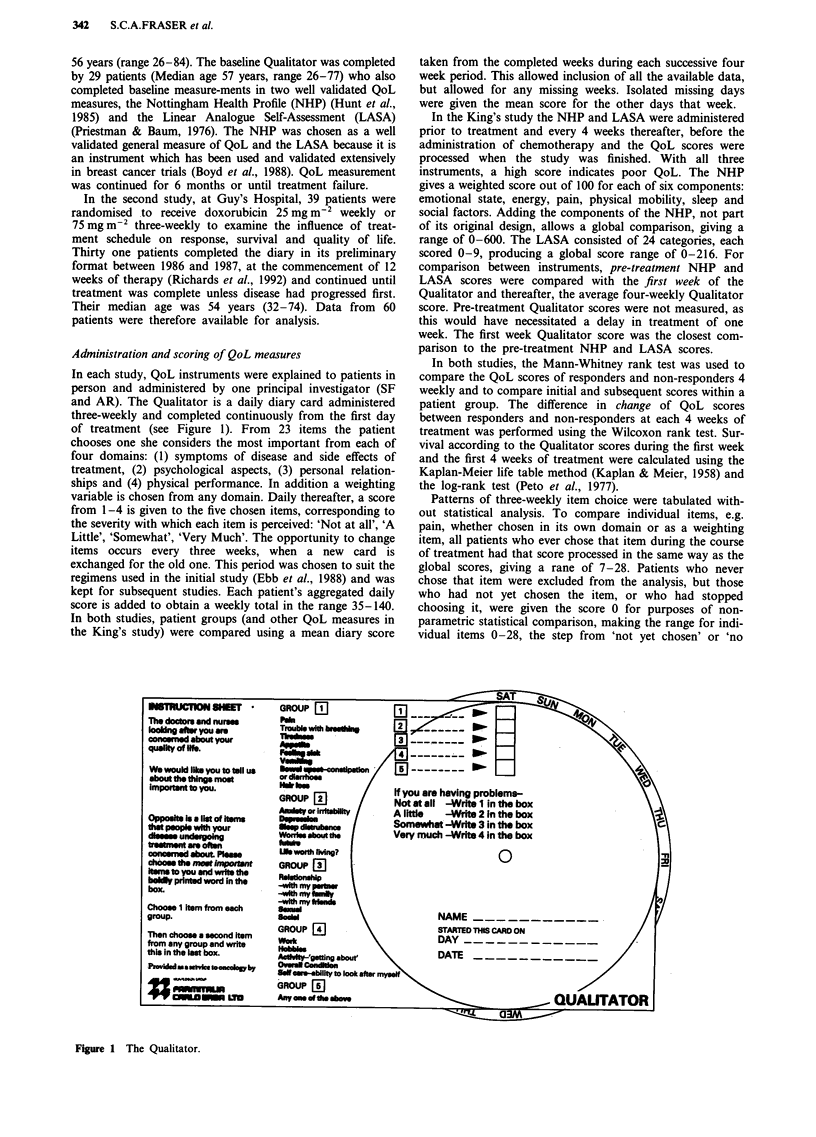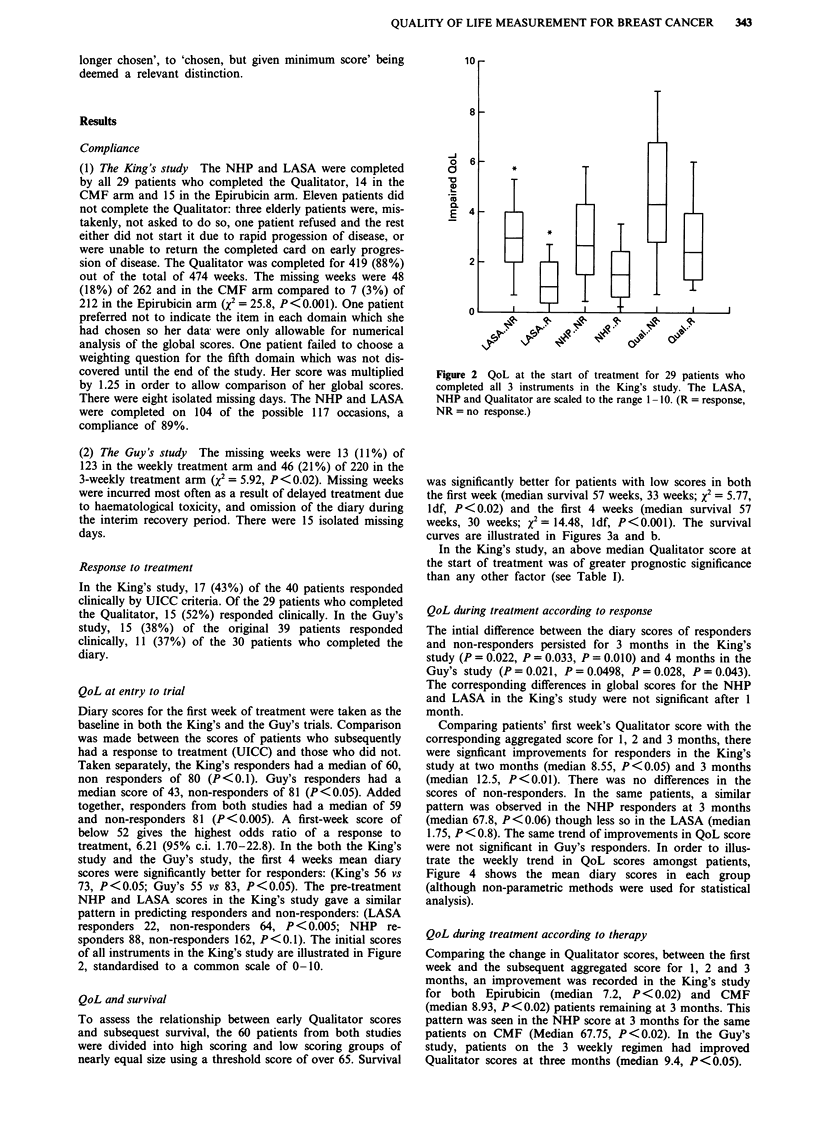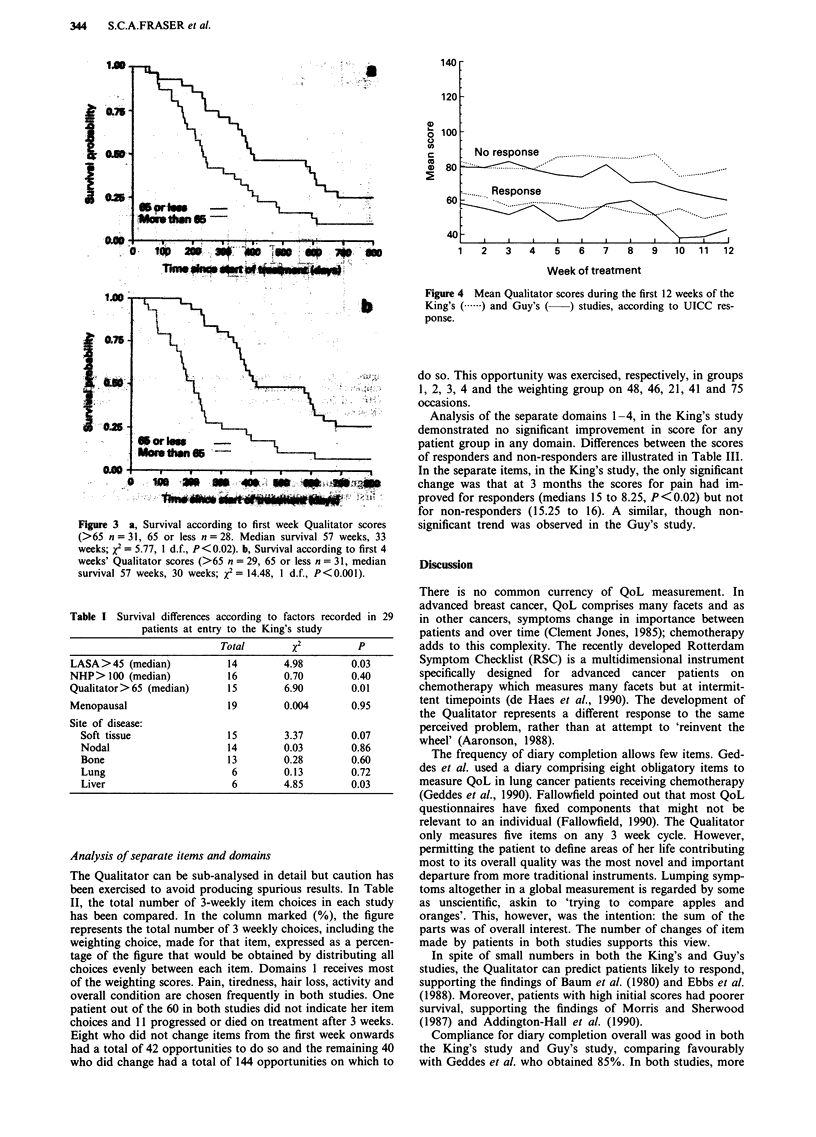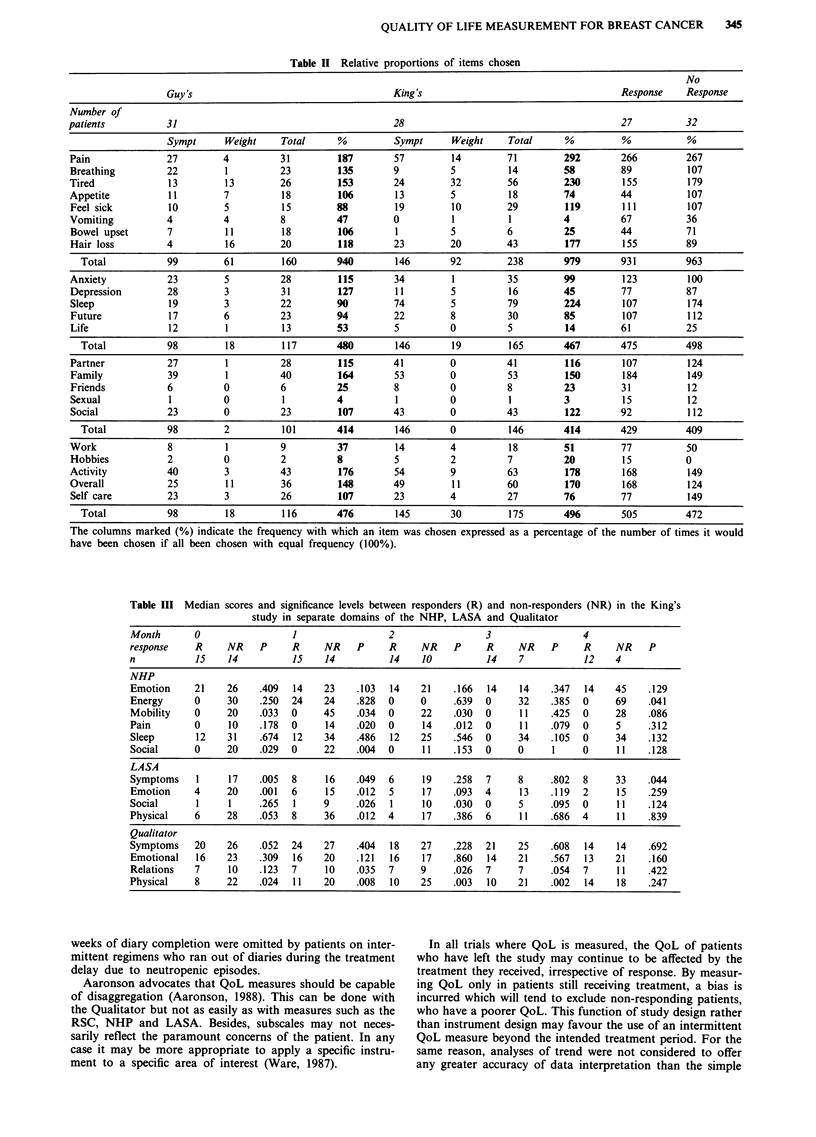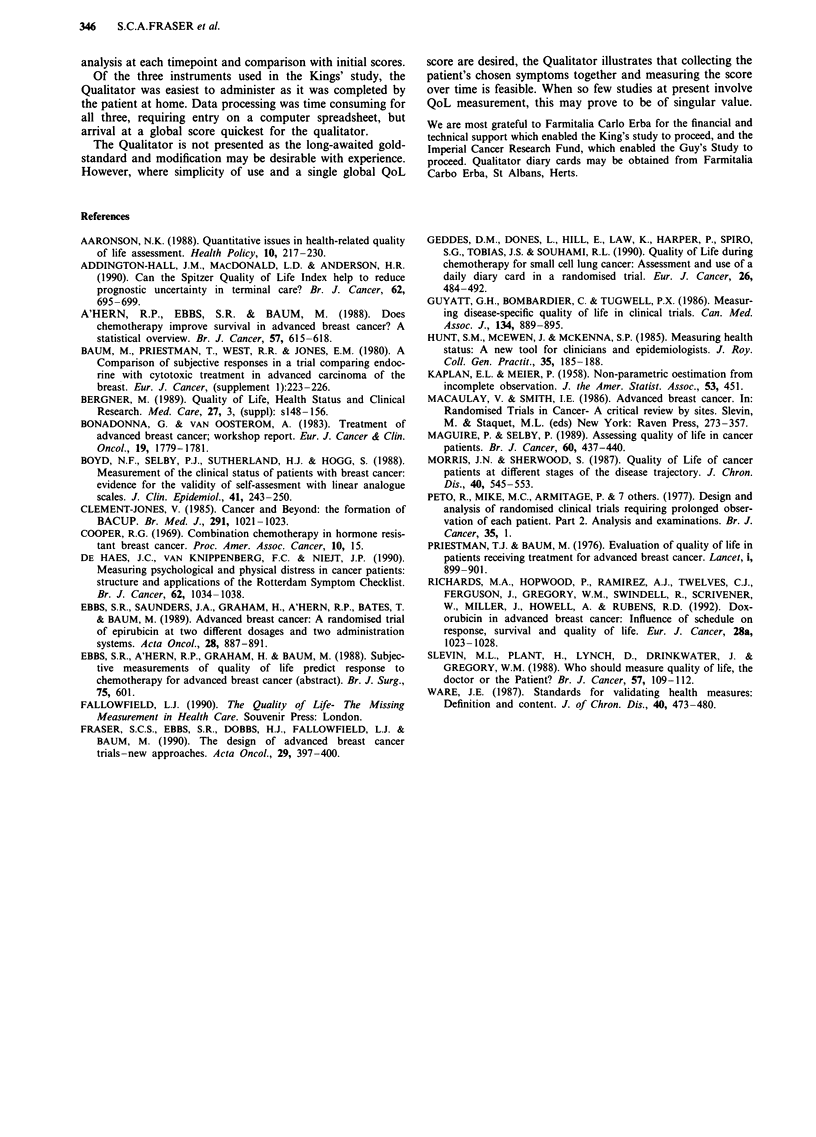# A daily diary for quality of life measurement in advanced breast cancer trials.

**DOI:** 10.1038/bjc.1993.62

**Published:** 1993-02

**Authors:** S. C. Fraser, A. J. Ramirez, S. R. Ebbs, L. J. Fallowfield, H. J. Dobbs, M. A. Richards, T. Bates, M. Baum

**Affiliations:** Department of Surgery, Kings College Hospital, London, UK.

## Abstract

The Qualitator is a daily diary card to measure Quality of Life, developed for use in chemotherapy trials for patients with advanced breast cancer. In a trial at King's College Hospital, 29 patients completed the Qualitator and their scores were compared with scores in the Linear Analogue Self-Assessment and Nottingham Health Profile taken four-weekly. In a separate study at Guy's Hospital, 31 patients completed the diary. The Qualitator offers accurate prognostic data regarding subsequent UICC response and survival and is simple to use.


Br. J. Cancer (1993), 67, 341–346 © Macmillan Press Ltd., 1993

### A daily diary for quality of life measurement in advanced breast cancer trials

S.C.A. Fraser[1], A.J. Ramirez[2], S.R. Ebbs[3], L.J. Fallowfield[4], H.J. Dobbs[5], M.A. Richards[2], T. Bates[6] & M. Baum[7]

[1]*Department of Surgery and* [5]*Department of Radiotherapy and Oncology, Kings College Hospital, Denmark Hill, London SE5 9RS;* [2]*Imperial Cancer Research Fund, Clinical Oncology Unit, Guy's Hospital, St. Thomas's Street, London SE1 9RT;* [3]*Department of Surgery, Mayday University Hospital, Mayday Road, Thornton Heath, Surrey CR7 7YE;* [4]*Cancer Research Campaign Communication and Counselling Research Centre, London Hospital Medical College, Turner St, London E1 2AD;* [6]*Department of Surgery, William Harvey Hospital, Ashford, Kent TN24 0LZ;* [7]*Institute of Cancer Research, Royal Marsden Hospital, Fulham Road, London SW3, UK.*

**Summary** The Qualitator is a daily diary card to measure Quality of Life, developed for use in chemotherapy trials for patients with advanced breast cancer. In a trial at King's College Hospital, 29 patients completed the Qualitator and their scores were compared with scores in the Linear Analogue Self-Assessment and Nottingham Health Profile taken four-weekly. In a separate study at Guy's Hospital, 31 patients completed the diary. The Qualitator offers accurate prognostic data regarding subsequent UICC response and survival and is simple to use.

The use of combination cytotoxic chemotherapy as palliation for patients with advanced breast cancer became established in the late 1960s (Cooper *et al.*, 1969). Few trials show a survival advantage for a particular regimen and only recently has an overall improvement in survival been associated with treatments giving higher response rates (A'Hern *et al.*, 1988). Although the aim of treatment is to improve the Quality of Life (QoL) of the patient, regimens are still compared on the basis of their response rate in patients where such measurements can be made. Side effects of chemotherapy such as alopecia, vomiting and lethargy are assumed to affect the QoL of the patients, but their objective measurement is a secondary aspect in most trials and subjective, patient derived, measurements are seldom made.

The simple technique of QoL measurement recording the patients' subjective symptoms using visual analogue scales was adapted for use in breast cancer patients by Priestman and Baum (1976). Since then, QoL measurement in cancer patients has been advocated widely (Maguire & Selby, 1989). However, in 1986, Macaulay and Smith reported that in a review of over 230 advanced breast cancer trials, in only two had overall QoL been measured. They added that assessment of the value of particular treatments should not rest upon response rate alone (Macaulay & Smith, 1986). So it is disappointing that during 1991, 15 years after Priestman and Baum's paper was written, of 48 studies of chemotherapy in advanced breast cancer listed in the index Medicus, we found only one which included QoL measurement. Many clinicians still prefer to rely upon their clinical judgement, although Slevin *et al.* found poor correlation between QoL measured by doctor and by patient (Slevin *et al.*, 1988). One problem may have been the QoL instruments on offer. Well-validated instruments did not include items about vomiting, nausea or hair loss and none was specific to breast cancer or chemotherapy. Moreover, QoL measurement is labour-intensive. We therefore addressed these problems.

In QoL measurement, a gold standard does not exist, and according to Bergner, is not desirable (Bergner, 1984). Instruments fall into two broad categories: multidimensional, designed to measure specific aspects of disease or treatment, and global, which give a single score for as broad a representation of QoL as possible. The former approach was chosen, to complement existing instruments, with weighting provided by allowing the patient to choose the items of relevance to her. To take account of the fluctuations which may be expected to occur in patients on chemotherapy, a diary format was adopted. Guidelines proposed in 1986 by Guyatt *et al.* (1986) were followed. A six stage process comprises item selection, item reduction, format design, pretesting, construct and test-retest reliability and finally validation. Items were amassed and distilled from all the QoL measures then available and others were added after consultation with a panel which included a psychologist, a surgeon, a GP and a nurse counsellor. The validation of the 'King's Diary' in it preliminary format was undertaken by Ebbs *et al.* during a trial comparing Epirubicin in two different doses and administration systems, in which 39 patients completed the initial form of the diary during their treatment (Ebbs *et al.*, 1989). This development process resulted in the 'Qualitator' and has been described previously (Fraser *et al.*, 1990). Validation has continued in two separate trials, described below.

To test the ability of the Qualitator to measure what it is purporting to measure, it is necessary to consider what is known so far about QoL in advanced breast cancer patients, and in cancer patients in general. Baum *et al.* reported that a response to chemotherapy improved QoL scores, especially for pain and insomnia (Baum *et al.*, 1980); Ebbs *et al.* reported that good pre-treatment QoL scores were associated with a subsequent response (Ebbs *et al.*, 1988). A relationship between poor QoL and poor survival was reported by Morris and Sherwood in a study of terminally ill patients (Morris & Sherwood, 1987). Later, Addington-Hall *et al.* used the Spitzer QoL Index to predict survival in 230 terminal patients (Addington-Hall *et al.*, 1990).

### Patients and methods

### *Patients*

Data were collected from two different studies, each with two arms. In the first, 40 patients with advanced breast cancer attending King's College Hospital and the William Harvey Hospital, Ashford, Kent were randomised to receive the standard 28 day cycle of CMF (Bonadonna *et al.*, 1983) or weekly Epirubicin 20 mg, for 6 months or until treatment failure between October 1988 and March 1990. The median age was

Correspondence: S.C.A. Fraser, Department of Surgery, Kings College Hospital, Denmark Hill, London SE5 9RS, UK.
Received 14 March, 1992; and in revised form 17 August 1992.

56 years (range 26–84). The baseline Qualitator was completed by 29 patients (Median age 57 years, range 26–77) who also completed baseline measure-ments in two well validated QoL measures, the Nottingham Health Profile (NHP) (Hunt *et al.*, 1985) and the Linear Analogue Self-Assessment (LASA) (Priestman & Baum, 1976). The NHP was chosen as a well validated general measure of QoL and the LASA because it is an instrument which has been used and validated extensively in breast cancer trials (Boyd *et al.*, 1988). QoL measurement was continued for 6 months or until treatment failure.

In the second study, at Guy's Hospital, 39 patients were randomised to receive doxorubicin 25 mg $m^{-2}$ weekly or 75 mg $m^{-2}$ three-weekly to examine the influence of treatment schedule on response, survival and quality of life. Thirty one patients completed the diary in its preliminary format between 1986 and 1987, at the commencement of 12 weeks of therapy (Richards *et al.*, 1992) and continued until treatment was complete unless disease had progressed first. Their median age was 54 years (32–74). Data from 60 patients were therefore available for analysis.

*Administration and scoring of QoL measures*

In each study, QoL instruments were explained to patients in person and administered by one principal investigator (SF and AR). The Qualitator is a daily diary card administered three-weekly and completed continuously from the first day of treatment (see Figure 1). From 23 items the patient chooses one she considers the most important from each of four domains: (1) symptoms of disease and side effects of treatment, (2) psychological aspects, (3) personal relationships and (4) physical performance. In addition a weighting variable is chosen from any domain. Daily thereafter, a score from 1–4 is given to the five chosen items, corresponding to the severity with which each item is perceived: 'Not at all', 'A Little', 'Somewhat', 'Very Much'. The opportunity to change items occurs every three weeks, when a new card is exchanged for the old one. This period was chosen to suit the regimens used in the initial study (Ebb *et al.*, 1988) and was kept for subsequent studies. Each patient's aggregated daily score is added to obtain a weekly total in the range 35–140. In both studies, patient groups (and other QoL measures in the King's study) were compared using a mean diary score taken from the completed weeks during each successive four week period. This allowed inclusion of all the available data, but allowed for any missing weeks. Isolated missing days were given the mean score for the other days that week.

In the King's study the NHP and LASA were administered prior to treatment and every 4 weeks thereafter, before the administration of chemotherapy and the QoL scores were processed when the study was finished. With all three instruments, a high score indicates poor QoL. The NHP gives a weighted score out of 100 for each of six components: emotional state, energy, pain, physical mobility, sleep and social factors. Adding the components of the NHP, not part of its original design, allows a global comparison, giving a range of 0–600. The LASA consisted of 24 categories, each scored 0–9, producing a global score range of 0–216. For comparison between instruments, *pre-treatment* NHP and LASA scores were compared with the *first week* of the Qualitator and thereafter, the average four-weekly Qualitator score. Pre-treatment Qualitator scores were not measured, as this would have necessitated a delay in treatment of one week. The first week Qualitator score was the closest comparison to the pre-treatment NHP and LASA scores.

In both studies, the Mann-Whitney rank test was used to compare the QoL scores of responders and non-responders 4 weekly and to compare initial and subsequent scores within a patient group. The difference in *change* of QoL scores between responders and non-responders at each 4 weeks of treatment was performed using the Wilcoxon rank test. Survival according to the Qualitator scores during the first week and the first 4 weeks of treatment were calculated using the Kaplan-Meier life table method (Kaplan & Meier, 1958) and the log-rank test (Peto *et al.*, 1977).

Patterns of three-weekly item choice were tabulated without statistical analysis. To compare individual items, e.g. pain, whether chosen in its own domain or as a weighting item, all patients who ever chose that item during the course of treatment had that score processed in the same way as the global scores, giving a rane of 7–28. Patients who never chose that item were excluded from the analysis, but those who had not yet chosen the item, or who had stopped choosing it, were given the score 0 for purposes of non-parametric statistical comparison, making the range for individual items 0–28, the step from 'not yet chosen' or 'no

**INSTRUCTION SHEET**

The doctors and nurses looking after you are concerned about your quality of life.

We would like you to tell us about the things most important to you.

Opposite is a list of items that people with your disease undergoing treatment are often concerned about. Please choose the *most important* items to you and write the **boldly** printed word in the box.

Choose 1 item from each group.

Then choose a second item from any group and write this in the last box.

Provided as a service to oncology by

FARMITALIA CARLO ERBA LTD

GROUP 1

**Pain**

Trouble with **breathing**

**Tiredness**

**Appetite**

**Feeling sick**

**Vomiting**

**Bowel upset**–constipation or diarrhoea

**Hair loss**

GROUP 2

**Anxiety** or irritability

**Depression**

**Sleep** distrubance

Worries about the **future**

**Life** worth living?

GROUP 3

Relationship

–with my **partner**

–with my **family**

–with my **friends**

**Sexual**

**Social**

GROUP 4

**Work**

**Hobbies**

**Activity**–'getting about'

**Overall Condition**

**Self care**–ability to look after myself

GROUP 5

Any one of the above

1 --------
2 --------
3 --------
4 --------
5 --------

SAT SUN MON TUE WED THU FRI SAT

If you are having problems–

Not at all –Write 1 in the box

A little –Write 2 in the box

Somewhat –Write 3 in the box

Very much –Write 4 in the box

NAME ____________
STARTED THIS CARD ON
DAY ____________
DATE ____________

QUALITATOR

**Figure 1** The Qualitator.

longer chosen', to 'chosen, but given minimum score' being deemed a relevant distinction.

### Results

### *Compliance*

(1) *The King's study* The NHP and LASA were completed by all 29 patients who completed the Qualitator, 14 in the CMF arm and 15 in the Epirubicin arm. Eleven patients did not complete the Qualitator: three elderly patients were, mistakenly, not asked to do so, one patient refused and the rest either did not start it due to rapid progession of disease, or were unable to return the completed card on early progression of disease. The Qualitator was completed for 419 (88%) out of the total of 474 weeks. The missing weeks were 48 (18%) of 262 and in the CMF arm compared to 7 (3%) of 212 in the Epirubicin arm ($\chi^2 = 25.8$, $P<0.001$). One patient preferred not to indicate the item in each domain which she had chosen so her data were only allowable for numerical analysis of the global scores. One patient failed to choose a weighting question for the fifth domain which was not discovered until the end of the study. Her score was multiplied by 1.25 in order to allow comparison of her global scores. There were eight isolated missing days. The NHP and LASA were completed on 104 of the possible 117 occasions, a compliance of 89%.

(2) *The Guy's study* The missing weeks were 13 (11%) of 123 in the weekly treatment arm and 46 (21%) of 220 in the 3-weekly treatment arm ($\chi^2 = 5.92$, $P<0.02$). Missing weeks were incurred most often as a result of delayed treatment due to haematological toxicity, and omission of the diary during the interim recovery period. There were 15 isolated missing days.

### *Response to treatment*

In the King's study, 17 (43%) of the 40 patients responded clinically by UICC criteria. Of the 29 patients who completed the Qualitator, 15 (52%) responded clinically. In the Guy's study, 15 (38%) of the original 39 patients responded clinically, 11 (37%) of the 30 patients who completed the diary.

### *QoL at entry to trial*

Diary scores for the first week of treatment were taken as the baseline in both the King's and the Guy's trials. Comparison was made between the scores of patients who subsequently had a response to treatment (UICC) and those who did not. Taken separately, the King's responders had a median of 60, non responders of 80 ($P<0.1$). Guy's responders had a median score of 43, non-responders of 81 ($P<0.05$). Added together, responders from both studies had a median of 59 and non-responders 81 ($P<0.005$). A first-week score of below 52 gives the highest odds ratio of a response to treatment, 6.21 (95% c.i. 1.70–22.8). In the both the King's study and the Guy's study, the first 4 weeks mean diary scores were significantly better for responders: (King's 56 *vs* 73, $P<0.05$; Guy's 55 *vs* 83, $P<0.05$). The pre-treatment NHP and LASA scores in the King's study gave a similar pattern in predicting responders and non-responders: (LASA responders 22, non-responders 64, $P<0.005$; NHP responders 88, non-responders 162, $P<0.1$). The initial scores of all instruments in the King's study are illustrated in Figure 2, standardised to a common scale of 0–10.

### *QoL and survival*

To assess the relationship between early Qualitator scores and subsequest survival, the 60 patients from both studies were divided into high scoring and low scoring groups of nearly equal size using a threshold score of over 65. Survival

**Figure 2** QoL at the start of treatment for 29 patients who completed all 3 instruments in the King's study. The LASA, NHP and Qualitator are scaled to the range 1–10. (R = response, NR = no response.)

was significantly better for patients with low scores in both the first week (median survival 57 weeks, 33 weeks; $\chi^2 = 5.77$, 1df, $P<0.02$) and the first 4 weeks (median survival 57 weeks, 30 weeks; $\chi^2 = 14.48$, 1df, $P<0.001$). The survival curves are illustrated in Figures 3a and b.

In the King's study, an above median Qualitator score at the start of treatment was of greater prognostic significance than any other factor (see Table I).

### *QoL during treatment according to response*

The intial difference between the diary scores of responders and non-responders persisted for 3 months in the King's study ($P = 0.022$, $P = 0.033$, $P = 0.010$) and 4 months in the Guy's study ($P = 0.021$, $P = 0.0498$, $P = 0.028$, $P = 0.043$). The corresponding differences in global scores for the NHP and LASA in the King's study were not significant after 1 month.

Comparing patients' first week's Qualitator score with the corresponding aggregated score for 1, 2 and 3 months, there were signficant improvements for responders in the King's study at two months (median 8.55, $P<0.05$) and 3 months (median 12.5, $P<0.01$). There was no differences in the scores of non-responders. In the same patients, a similar pattern was observed in the NHP responders at 3 months (median 67.8, $P<0.06$) though less so in the LASA (median 1.75, $P<0.8$). The same trend of improvements in QoL score were not significant in Guy's responders. In order to illustrate the weekly trend in QoL scores amongst patients, Figure 4 shows the mean diary scores in each group (although non-parametric methods were used for statistical analysis).

### *QoL during treatment according to therapy*

Comparing the change in Qualitator scores, between the first week and the subsequent aggregated score for 1, 2 and 3 months, an improvement was recorded in the King's study for both Epirubicin (median 7.2, $P<0.02$) and CMF (median 8.93, $P<0.02$) patients remaining at 3 months. This pattern was seen in the NHP score at 3 months for the same patients on CMF (Median 67.75, $P<0.02$). In the Guy's study, patients on the 3 weekly regimen had improved Qualitator scores at three months (median 9.4, $P<0.05$).

**Figure 3** **a**, Survival according to first week Qualitator scores (>65 $n = 31$, 65 or less $n = 28$. Median survival 57 weeks, 33 weeks; $\chi^2 = 5.77$, 1 d.f., $P < 0.02$). **b**, Survival according to first 4 weeks' Qualitator scores (>65 $n = 29$, 65 or less $n = 31$, median survival 57 weeks, 30 weeks; $\chi^2 = 14.48$, 1 d.f., $P < 0.001$).

**Table I** Survival differences according to factors recorded in 29 patients at entry to the King's study

| | *Total* | $\chi^2$ | *P* |
|---|---|---|---|
| LASA>45 (median) | 14 | 4.98 | 0.03 |
| NHP>100 (median) | 16 | 0.70 | 0.40 |
| Qualitator>65 (median) | 15 | 6.90 | 0.01 |
| Menopausal | 19 | 0.004 | 0.95 |
| Site of disease: | | | |
| Soft tissue | 15 | 3.37 | 0.07 |
| Nodal | 14 | 0.03 | 0.86 |
| Bone | 13 | 0.28 | 0.60 |
| Lung | 6 | 0.13 | 0.72 |
| Liver | 6 | 4.85 | 0.03 |

*Analysis of separate items and domains*

The Qualitator can be sub-analysed in detail but caution has been exercised to avoid producing spurious results. In Table II, the total number of 3-weekly item choices in each study has been compared. In the column marked (%), the figure represents the total number of 3 weekly choices, including the weighting choice, made for that item, expressed as a percentage of the figure that would be obtained by distributing all choices evenly between each item. Domains 1 receives most of the weighting scores. Pain, tiredness, hair loss, activity and overall condition are chosen frequently in both studies. One patient out of the 60 in both studies did not indicate her item choices and 11 progressed or died on treatment after 3 weeks. Eight who did not change items from the first week onwards had a total of 42 opportunities to do so and the remaining 40 who did change had a total of 144 opportunities on which to do so. This opportunity was exercised, respectively, in groups 1, 2, 3, 4 and the weighting group on 48, 46, 21, 41 and 75 occasions.

**Figure 4** Mean Qualitator scores during the first 12 weeks of the King's (······) and Guy's (——) studies, according to UICC response.

Analysis of the separate domains 1–4, in the King's study demonstrated no significant improvement in score for any patient group in any domain. Differences between the scores of responders and non-responders are illustrated in Table III. In the separate items, in the King's study, the only significant change was that at 3 months the scores for pain had improved for responders (medians 15 to 8.25, $P < 0.02$) but not for non-responders (15.25 to 16). A similar, though non-significant trend was observed in the Guy's study.

### Discussion

There is no common currency of QoL measurement. In advanced breast cancer, QoL comprises many facets and as in other cancers, symptoms change in importance between patients and over time (Clement Jones, 1985); chemotherapy adds to this complexity. The recently developed Rotterdam Symptom Checklist (RSC) is a multidimensional instrument specifically designed for advanced cancer patients on chemotherapy which measures many facets but at intermittent timepoints (de Haes *et al.*, 1990). The development of the Qualitator represents a different response to the same perceived problem, rather than at attempt to 'reinvent the wheel' (Aaronson, 1988).

The frequency of diary completion allows few items. Geddes *et al.* used a diary comprising eight obligatory items to measure QoL in lung cancer patients receiving chemotherapy (Geddes *et al.*, 1990). Fallowfield pointed out that most QoL questionnaires have fixed components that might not be relevant to an individual (Fallowfield, 1990). The Qualitator only measures five items on any 3 week cycle. However, permitting the patient to define areas of her life contributing most to its overall quality was the most novel and important departure from more traditional instruments. Lumping symptoms altogether in a global measurement is regarded by some as unscientific, askin to 'trying to compare apples and oranges'. This, however, was the intention: the sum of the parts was of overall interest. The number of changes of item made by patients in both studies supports this view.

In spite of small numbers in both the King's and Guy's studies, the Qualitator can predict patients likely to respond, supporting the findings of Baum *et al.* (1980) and Ebbs *et al.* (1988). Moreover, patients with high initial scores had poorer survival, supporting the findings of Morris and Sherwood (1987) and Addington-Hall *et al.* (1990).

Compliance for diary completion overall was good in both the King's study and Guy's study, comparing favourably with Geddes *et al.* who obtained 85%. In both studies, more

**Table II** Relative proportions of items chosen

| | *Guy's* | | | | *King's* | | | | *Response* | *No Response* |
|---|---|---|---|---|---|---|---|---|---|---|
| *Number of patients* | *31* | | | | *28* | | | | *27* | *32* |
| | *Sympt* | *Weight* | *Total* | *%* | *Sympt* | *Weight* | *Total* | *%* | *%* | *%* |
| Pain | 27 | 4 | 31 | **187** | 57 | 14 | 71 | **292** | 266 | 267 |
| Breathing | 22 | 1 | 23 | **135** | 9 | 5 | 14 | **58** | 89 | 107 |
| Tired | 13 | 13 | 26 | **153** | 24 | 32 | 56 | **230** | 155 | 179 |
| Appetite | 11 | 7 | 18 | **106** | 13 | 5 | 18 | **74** | 44 | 107 |
| Feel sick | 10 | 5 | 15 | **88** | 19 | 10 | 29 | **119** | 111 | 107 |
| Vomiting | 4 | 4 | 8 | **47** | 0 | 1 | 1 | **4** | 67 | 36 |
| Bowel upset | 7 | 11 | 18 | **106** | 1 | 5 | 6 | **25** | 44 | 71 |
| Hair loss | 4 | 16 | 20 | **118** | 23 | 20 | 43 | **177** | 155 | 89 |
| Total | 99 | 61 | 160 | **940** | 146 | 92 | 238 | **979** | 931 | 963 |
| Anxiety | 23 | 5 | 28 | **115** | 34 | 1 | 35 | **99** | 123 | 100 |
| Depression | 28 | 3 | 31 | **127** | 11 | 5 | 16 | **45** | 77 | 87 |
| Sleep | 19 | 3 | 22 | **90** | 74 | 5 | 79 | **224** | 107 | 174 |
| Future | 17 | 6 | 23 | **94** | 22 | 8 | 30 | **85** | 107 | 112 |
| Life | 12 | 1 | 13 | **53** | 5 | 0 | 5 | **14** | 61 | 25 |
| Total | 98 | 18 | 117 | **480** | 146 | 19 | 165 | **467** | 475 | 498 |
| Partner | 27 | 1 | 28 | **115** | 41 | 0 | 41 | **116** | 107 | 124 |
| Family | 39 | 1 | 40 | **164** | 53 | 0 | 53 | **150** | 184 | 149 |
| Friends | 6 | 0 | 6 | **25** | 8 | 0 | 8 | **23** | 31 | 12 |
| Sexual | 1 | 0 | 1 | **4** | 1 | 0 | 1 | **3** | 15 | 12 |
| Social | 23 | 0 | 23 | **107** | 43 | 0 | 43 | **122** | 92 | 112 |
| Total | 98 | 2 | 101 | **414** | 146 | 0 | 146 | **414** | 429 | 409 |
| Work | 8 | 1 | 9 | **37** | 14 | 4 | 18 | **51** | 77 | 50 |
| Hobbies | 2 | 0 | 2 | **8** | 5 | 2 | 7 | **20** | 15 | 0 |
| Activity | 40 | 3 | 43 | **176** | 54 | 9 | 63 | **178** | 168 | 149 |
| Overall | 25 | 11 | 36 | **148** | 49 | 11 | 60 | **170** | 168 | 124 |
| Self care | 23 | 3 | 26 | **107** | 23 | 4 | 27 | **76** | 77 | 149 |
| Total | 98 | 18 | 116 | **476** | 145 | 30 | 175 | **496** | 505 | 472 |

The columns marked (%) indicate the frequency with which an item was chosen expressed as a percentage of the number of times it would have been chosen if all been chosen with equal frequency (100%).

**Table III** Median scores and significance levels between responders (R) and non-responders (NR) in the King's study in separate domains of the NHP, LASA and Qualitator

| *Month* | *0* | | | *1* | | | *2* | | | *3* | | | *4* | | |
|---|---|---|---|---|---|---|---|---|---|---|---|---|---|---|---|
| *response* | *R* | *NR* | *P* | *R* | *NR* | *P* | *R* | *NR* | *P* | *R* | *NR* | *P* | *R* | *NR* | *P* |
| *n* | *15* | *14* | | *15* | *14* | | *14* | *10* | | *14* | *7* | | *12* | *4* | |
| *NHP* | | | | | | | | | | | | | | | |
| Emotion | 21 | 26 | .409 | 14 | 23 | .103 | 14 | 21 | .166 | 14 | 14 | .347 | 14 | 45 | .129 |
| Energy | 0 | 30 | .250 | 24 | 24 | .828 | 0 | 0 | .639 | 0 | 32 | .385 | 0 | 69 | .041 |
| Mobility | 0 | 20 | .033 | 0 | 45 | .034 | 0 | 22 | .030 | 0 | 11 | .425 | 0 | 28 | .086 |
| Pain | 0 | 10 | .178 | 0 | 14 | .020 | 0 | 14 | .012 | 0 | 11 | .079 | 0 | 5 | .312 |
| Sleep | 12 | 31 | .674 | 12 | 34 | .486 | 12 | 25 | .546 | 0 | 34 | .105 | 0 | 34 | .132 |
| Social | 0 | 20 | .029 | 0 | 22 | .004 | 0 | 11 | .153 | 0 | 0 | 1 | 0 | 11 | .128 |
| *LASA* | | | | | | | | | | | | | | | |
| Symptoms | 1 | 17 | .005 | 8 | 16 | .049 | 6 | 19 | .258 | 7 | 8 | .802 | 8 | 33 | .044 |
| Emotion | 4 | 20 | .001 | 6 | 15 | .012 | 5 | 17 | .093 | 4 | 13 | .119 | 2 | 15 | .259 |
| Social | 1 | 1 | .265 | 1 | 9 | .026 | 1 | 10 | .030 | 0 | 5 | .095 | 0 | 11 | .124 |
| Physical | 6 | 28 | .053 | 8 | 36 | .012 | 4 | 17 | .386 | 6 | 11 | .686 | 4 | 11 | .839 |
| *Qualitator* | | | | | | | | | | | | | | | |
| Symptoms | 20 | 26 | .052 | 24 | 27 | .404 | 18 | 27 | .228 | 21 | 25 | .608 | 14 | 14 | .692 |
| Emotional | 16 | 23 | .309 | 16 | 20 | .121 | 16 | 17 | .860 | 14 | 21 | .567 | 13 | 21 | .160 |
| Relations | 7 | 10 | .123 | 7 | 10 | .035 | 7 | 9 | .026 | 7 | 7 | .054 | 7 | 11 | .422 |
| Physical | 8 | 22 | .024 | 11 | 20 | .008 | 10 | 25 | .003 | 10 | 21 | .002 | 14 | 18 | .247 |

weeks of diary completion were omitted by patients on intermittent regimens who ran out of diaries during the treatment delay due to neutropenic episodes.

Aaronson advocates that QoL measures should be capable of disaggregation (Aaronson, 1988). This can be done with the Qualitator but not as easily as with measures such as the RSC, NHP and LASA. Besides, subscales may not necessarily reflect the paramount concerns of the patient. In any case it may be more appropriate to apply a specific instrument to a specific area of interest (Ware, 1987).

In all trials where QoL is measured, the QoL of patients who have left the study may continue to be affected by the treatment they received, irrespective of response. By measuring QoL only in patients still receiving treatment, a bias is incurred which will tend to exclude non-responding patients, who have a poorer QoL. This function of study design rather than instrument design may favour the use of an intermittent QoL measure beyond the intended treatment period. For the same reason, analyses of trend were not considered to offer any greater accuracy of data interpretation than the simple analysis at each timepoint and comparison with initial scores.

Of the three instruments used in the Kings' study, the Qualitator was easiest to administer as it was completed by the patient at home. Data processing was time consuming for all three, requiring entry on a computer spreadsheet, but arrival at a global score quickest for the qualitator.

The Qualitator is not presented as the long-awaited gold-standard and modification may be desirable with experience. However, where simplicity of use and a single global QoL score are desired, the Qualitator illustrates that collecting the patient's chosen symptoms together and measuring the score over time is feasible. When so few studies at present involve QoL measurement, this may prove to be of singular value.

We are most grateful to Farmitalia Carlo Erba for the financial and technical support which enabled the King's study to proceed, and the Imperial Cancer Research Fund, which enabled the Guy's Study to proceed. Qualitator diary cards may be obtained from Farmitalia Carbo Erba, St Albans, Herts.

### References

AARONSON, N.K. (1988). Quantitative issues in health-related quality of life assessment. *Health Policy*, **10**, 217–230.

ADDINGTON-HALL, J.M., MACDONALD, L.D. & ANDERSON, H.R. (1990). Can the Spitzer Quality of Life Index help to reduce prognostic uncertainty in terminal care? *Br. J. Cancer*, **62**, 695–699.

A'HERN, R.P., EBBS, S.R. & BAUM, M. (1988). Does chemotherapy improve survival in advanced breast cancer? A statistical overview. *Br. J. Cancer*, **57**, 615–618.

BAUM, M., PRIESTMAN, T., WEST, R.R. & JONES, E.M. (1980). A Comparison of subjective responses in a trial comparing endocrine with cytotoxic treatment in advanced carcinoma of the breast. *Eur. J. Cancer*, (supplement 1):223–226.

BERGNER, M. (1989). Quality of Life, Health Status and Clinical Research. *Med. Care*, **27**, 3, (suppl): s148–156.

BONADONNA, G. & VAN OOSTEROM, A. (1983). Treatment of advanced breast cancer; workshop report. *Eur. J. Cancer & Clin. Oncol.*, **19**, 1779–1781.

BOYD, N.F., SELBY, P.J., SUTHERLAND, H.J. & HOGG, S. (1988). Measurement of the clinical status of patients with breast cancer: evidence for the validity of self-assesment with linear analogue scales. *J. Clin. Epidemiol.*, **41**, 243–250.

CLEMENT-JONES, V. (1985). Cancer and Beyond: the formation of BACUP. *Br. Med. J.*, **291**, 1021–1023.

COOPER, R.G. (1969). Combination chemotherapy in hormone resistant breast cancer. *Proc. Amer. Assoc. Cancer*, **10**, 15.

DE HAES, J.C., VAN KNIPPENBERG, F.C. & NIEJT, J.P. (1990). Measuring psychological and physical distress in cancer patients: structure and applications of the Rotterdam Symptom Checklist. *Br. J. Cancer*, **62**, 1034–1038.

EBBS, S.R., SAUNDERS, J.A., GRAHAM, H., A'HERN, R.P., BATES, T. & BAUM, M. (1989). Advanced breast cancer: A randomised trial of epirubicin at two different dosages and two administration systems. *Acta Oncol.*, **28**, 887–891.

EBBS, S.R., A'HERN, R.P., GRAHAM, H. & BAUM, M. (1988). Subjective measurements of quality of life predict response to chemotherapy for advanced breast cancer (abstract). *Br. J. Surg.*, **75**, 601.

FALLOWFIELD, L.J. (1990). *The Quality of Life- The Missing Measurement in Health Care*. Souvenir Press: London.

FRASER, S.C.S., EBBS, S.R., DOBBS, H.J., FALLOWFIELD, L.J. & BAUM, M. (1990). The design of advanced breast cancer trials–new approaches. *Acta Oncol.*, **29**, 397–400.

GEDDES, D.M., DONES, L., HILL, E., LAW, K., HARPER, P., SPIRO, S.G., TOBIAS, J.S. & SOUHAMI, R.L. (1990). Quality of Life during chemotherapy for small cell lung cancer: Assessment and use of a daily diary card in a randomised trial. *Eur. J. Cancer*, **26**, 484–492.

GUYATT, G.H., BOMBARDIER, C. & TUGWELL, P.X. (1986). Measuring disease-specific quality of life in clinical trials. *Can. Med. Assoc. J.*, **134**, 889–895.

HUNT, S.M., MCEWEN, J. & MCKENNA, S.P. (1985). Measuring health status: A new tool for clinicians and epidemiologists. *J. Roy. Coll. Gen. Practit.*, **35**, 185–188.

KAPLAN, E.L. & MEIER, P. (1958). Non-parametric oestimation from incomplete observation. *J. the Amer. Statist. Assoc.*, **53**, 451.

MACAULAY, V. & SMITH, I.E. (1986). Advanced breast cancer. In: Randomised Trials in Cancer- A critical review by sites. Slevin, M. & Staquet, M.L. (eds) New York: Raven Press, 273–357.

MAGUIRE, P. & SELBY, P. (1989). Assessing quality of life in cancer patients. *Br. J. Cancer*, **60**, 437–440.

MORRIS, J.N. & SHERWOOD, S. (1987). Quality of Life of cancer patients at different stages of the disease trajectory. *J. Chron. Dis.*, **40**, 545–553.

PETO, R., MIKE, M.C., ARMITAGE, P. & 7 others. (1977). Design and analysis of randomised clinical trials requiring prolonged observation of each patient. Part 2. Analysis and examinations. *Br. J. Cancer*, **35**, 1.

PRIESTMAN, T.J. & BAUM, M. (1976). Evaluation of quality of life in patients receiving treatment for advanced breast cancer. *Lancet*, **i**, 899–901.

RICHARDS, M.A., HOPWOOD, P., RAMIREZ, A.J., TWELVES, C.J., FERGUSON, J., GREGORY, W.M., SWINDELL, R., SCRIVENER, W., MILLER, J., HOWELL, A. & RUBENS, R.D. (1992). Doxorubicin in advanced breast cancer: Influence of schedule on response, survival and quality of life. *Eur. J. Cancer*, **28a**, 1023–1028.

SLEVIN, M.L., PLANT, H., LYNCH, D., DRINKWATER, J. & GREGORY, W.M. (1988). Who should measure quality of life, the doctor or the Patient? *Br. J. Cancer*, **57**, 109–112.

WARE, J.E. (1987). Standards for validating health measures: Definition and content. *J. of Chron. Dis.*, **40**, 473–480.